# Association of FTO common variant (rs9939609) with body fat in Turkish individuals

**DOI:** 10.1186/s12944-019-1160-y

**Published:** 2019-12-06

**Authors:** Duygu AĞAGÜNDÜZ, Makbule GEZMEN-KARADAĞ

**Affiliations:** Department of Nutrition and Dietetics, Emniyet Mahallesi, Muammer Yaşar Bostancı Caddesi, Gazi University Faculty of Health Sciences,, No:16, 06500 Beşevler, Ankara, Turkey

**Keywords:** FTO, rs9939609, Adiposity

## Abstract

**Background:**

Variations in the fat mass and obesity-associated (FTO) gene (16q12.2) are associated with obesity in some populations. This study aimed to determine the relationship between FTO gene polymorphism and adiposity&related markers in Turkish adults was aimed.

**Methods:**

The present study included 200 participants aged 18–65 years, who were genotyped for variants of the FTO gene (rs9939609). Anthropometric measurements were performed. Body compositions were analyzed with Tanita BC 545 N Inner ScanTM. Infrared analyzer (VISCANTM) was also used to determinate the degree of abdominal fat. Body mass index (BMI), body adiposity index (BAI) and lipid accumulation products (LAP) index which are used in body fat estimation were calculated. Body fat amounts were classified using gender-based cut-offs. Odds ratio (OR) and 95% confidence interval (CI) were calculated to determine the risk of having a high body fat amount associated with the risk allele.

**Results:**

The frequency of the rs9939609 AA genotype was 19.0%, which was 42.5% for the AT genotype and 38.5% for the TT genotype (wild type). AA genotype was found to be higher in females than in males (26.0 and 12.0%, respectively). The total body fat amount of the individuals with AA genotype was high (28.5 ± 9.25%) compared to AT (27.0 ± 10.31%) and TT (23.7 ± 10.62%) genotype (*p* < 0.05). However, there was no difference in abdominal fat amounts (%) (AA:38.6%, AT:36.2%, TT:33.7%), internal fat levels and waist/hip ratios (*p* > 0.05). Significance of association between FTO genotypes and total body fat (%) was retained after adjustment for BMI and gender as well. BMI, LAP, and BAI index values were not different between different genotypes in all individuals and different genders (*p* > 0.05).

**Conclusion:**

Our study supports that the FTO rs9939609 polymorphism is associated with fat accumulation in the whole body without being associated with abdominal fat accumulation in Turkish adults.

## Background

Obesity is defined as a “multi-metabolic” and “hormonal” disease and it is affected by sociodemographic, environmental, genetic and many physiological factors [1]. In obesity which is a multifactorial disease, genetic factors are estimated to account Obesity is defined as a “multi-metabolic” and “hormonal” disease and it is affected by sociodemographic, environmental, genetic and many physiological factors [1]. In obesity which is a multifactorial disease, genetic factors are estimated to account for 40–70% [2].

There are two approaches to the identification of genes affecting obesity and chronic diseases: “candidate gene association studies” and “genome-wide association studies (GWAS)” [3]. In this context, a 2007 GWAS showed that fat mass and an obesity-related gene (FTO) variant are associated with type 2 diabetes and the gene’s effect on obesity can regulate this relationship [4].

FTO is a gene encoding a polypeptide with 9 exons and 505 amino acids. The protein encoded by this gene is a Fe ^+ 2^ and 2-oxoglutarate (α-ketoglutarate) dependent dioxygenase enzyme that repairs alkylated deoxyribonucleic acid (DNA) and ribonucleic acid (RNA) by oxidative demethylation. FTO is expressed in a wide range in various tissues, including the hypothalamus and pituitary where its levels are especially high [5]. Although its physiological role is not well defined, it is thought to have a role in the hypothalamic control of energy intake, in the adipogenesis with energy consumption and mitochondrial function of skeletal muscle due to the tissues in which it is expressed [6].

Several GWAS studies have shown that variations in the FTO gene are associated with obesity [7, 8]. Especially the single nucleotide polymorphism (SNP) (rs9939609) and mutations in the first intron of the FTO gene (in the non-protein coding area) have been reported to be associated with obesity in children and adult populations [9]. Similar to these results, it was found that the body weights were 3–4 kg more and the obesity risk was 1.67 times higher, in those who were homozygous (AA genotype) for the risk allele than those who were not [10]. In another study, individuals with rs9939609 AA genotype were found to have higher body mass index (BMI) as well as fat mass and waist circumference than individuals with AT and TT genotypes [11]. Some studies in the literature show the contribution of rs17817449, rs3751812, rs1421085 and rs9930506, rs7202116 SNPs to the association between FTO and childhood/adult obesity [4, 11–13].

The effect of the FTO gene on nutrition and thus energy intake is among the subjects which are drawing attention. In a study, genetic variations (rs17817449 and rs142085) occurring in the loci of the FTO gene were reported to contribute to the etiology of obesity by increasing the level of plasma leptin and causing the insulin resistance [14]. In another study, it was reported that the messenger RNA (mRNA) level of acyl ghrelin was higher in individuals with FTO gene AA genotype compared to individuals with TT genotype [15].

Although in many recently conducted GWAS, one of the major causes of obesity has been reported to be the FTO gene polymorphism (rs9939609) [7, 8, 10], scarcely any studies have determined the frequency of the polymorphism of the FTO gene in the Turkish population, and they have conflicting results. Besides, it is controversial whether the polymorphism of this gene contributes to adiposity and by which mechanisms. In this study, the determination of the relationship between FTO gene polymorphism and adiposity&related markers in Turkish adults was aimed.

## Methods

### Subjects and setting

The present study was conducted on 100 females and 100 males, a total of 200 volunteered Turkish adults (18–65 years) who had different BMI values (underweight:50 individuals, normal weight:50 individuals; overweight:50 individuals and obese:50 individuals) and admitted to the Gazi University’s Nutrition and Diet Center. Homogeneity in age, gender, and BMI have been cared. Those who were relatives, those of different ethnic backgrounds, those with cancer, liver and kidney failure, hypothyroidism and hyperthyroidism, Type 2 diabetes mellitus, those with alcohol and substance addiction, those who were pregnant and breastfeeding, and individuals whom with concerns in adapting to the research were not included in the study.

### Questionnaire form

General characteristics, health histories and lifestyle habits of individuals were determined with a questionnaire through the face-to-face interview method.

### The international physical activity questionnaire - short form (IPAQ-SF)

the IPAQ short form whose validity and reliability studies have been performed in Turkey was used to determine the physical activity levels of the individuals [16]. The minute and frequency (day) values of the related activity group were multiplied by the metabolic equivalents (MET) values. The obtained product values were totalized in the final stage and the total physical activity value was obtained. The values obtained from the calculations (< 600 MET- minute/week, 600–3000 MET-minute/week,> 3000 MET-minute/week) were classified [16, 17].

### Measurements

#### Anthropometric measurements

Anthropometric parameters were directly measured by standard protocols. Anthropometric measurements such as body weight (kg), waist circumference (cm) and hip circumference (cm) were obtained and waist/hip ratios were calculated.

The body weights were measured using the Tanita BC 545 N Inner Scan (Balance TM) while the subjects were hungry and wearing light clothes. Height was measured (cm) with feet close together and the head in Frankfort plane with a stadiometer with 0.1-cm accuracy. The BMI was calculated using the “body weight / height2” (kg/m^2^) formula [18]. According to the BMI classification of the World Health Organization (WHO), The BMI values of the participants were grouped into four categories: underweight (BMI < 18.5 kg/m^2^), normal weight (18.5–24.9 kg/m^2^), overweight (25.0 ≤ BMI kg/m^2^), and obese (30.0 ≤ BMI kg/m^2^) [18].

#### Body composition analysis and adiposity indexes

Body composition measurements were performed using Tanita BC 545 N Inner Scan TM with bioelectrical impedance analysis (BIA). Total body fat amounts were classified as “non-high” and “high” by using cut-off values of Lee and Nieman’s gender-based amount of body fat (%) [19]. To determine the degree of internal fat level and central fat percentage (%), a portable VISCAN Abdominal Fat Analyzer TM based on an infrared method was used.

In addition, “body adiposity index (BAI)” of individuals; (Hip circumference (cm) / height (m)1.5)-18] was calculated. The BAI value obtained from the calculation can predict the amount of body fat (%) without any correction in adult males and females of different ethnic origin [20]. “Lipid accumulation products (LAP) index” of individuals [Male = (Waist circumference-65) x serum triglyceride level (TG, mmoL); female: (Waist circumference-58) x serum TG level (mmoL)] were also calculated [21]. In this calculation, the value of the waist circumference in cm was used.

#### Serum biochemical measurements

Morning blood samples of individuals were taken after overnight fasting (8–10 h) following the dinner. The blood lipid profile of individuals was analyzed in the biochemistry laboratory to be used to calculate fasting insulin (uIU/mL) and lipid accumulation products (LAP) index.

To determine the fasting serum leptin and ghrelin levels, approximately 7 mL of blood samples were taken to the vacuum gel tube and the blood samples were centrifuged at 1000×g for 15 min with the YUDA 800D® brand device. In the next step, the supernatant portion was separated and transferred to 1.5 mL of Eppendorf. Serum samples were stored at − 32 0C in the freezer until the time of analysis. The samples were analyzed in duplicates. Leptin and ghrelin analysis in serum samples was performed by the Enzyme-linked immunosorbent assay (ELISA) method using Human Leptin-LEP ELISA Kit (CUSABIO®) and Human Ghrelin-GHRL ELISA Kit (CUSABIO®). The quantitative sandwich ELISA method was used in the analysis. The results obtained were expressed in ng/mL for leptin and in pg/mL for total ghrelin.

#### DNA extraction and genotyping

For the genetic analysis, 3 mL of blood was collected from the individuals into tubes containing ethylenediaminetetraacetic acid (EDTA) and blood samples were stored at − 80 °C until the time of analysis. For isolation of DNA from blood samples in EDTA tubes obtained from individuals, GF-1 Blood DNA Extraction Kit (Vivantis®, Malaysia) was used. In the stage after isolation, the DNAs were stored in nuclease-free microcentrifuge tubes with 1.5 mL volume. Isolated genomic DNA concentration and purity were measured on a NanoDrop™ UV spectrophotometer (Thermo Fisher Scientific®, USA).

Real-time polymerase chain reaction (PCR) (ABI 7500 FAST, USA) with hydrolysis based probe were used to determine the intronic FTO gene rs9939609 (T/A) SNPs. In the genetic analysis, 3 min of pre-denaturation at 95 °C (first denaturation), 10 s of denaturation at 10 °C (DNA chain opening), 30 s of annealing at 59 °C (attachment/bonding of the primers to the opened DNA chain) and 5 s of primary extension at 72 °C were realized as the PCR protocol. This cycle was repeated a total of 40 times. After the PCR cycle, the cooling process was started and the products were stored at 40 °C for 1 min with PCR.

In the present study, SNP genotyping was performed by the hydrolysis probe method. The rs9939609 probe (T) Fam and rs9939609 probe (A) Hex used in the study are marked with fluorescent dyes. Black Hole Quencher (BHQ), a chemical capable of absorbing the free fluorescent light defined as a quencher, was added to the 3′ terminals and marked. When the Fam radiation attached to Allele T, and the Hex radiation attached to Allele A, an increase is not provided. If both probe radiations occur, the presence of the two alleles was understood. In the final stage, the individuals were classified into 3 groups according to the condition of having AA, AT, TT genotypes, based on the polymorphisms of rs9939609 of the FTO gene. In the literature, the “A allele” is defined as the risk allele for obesity in terms of the FTO gene rs9939609 polymorphism [22]. In this scope, AA genotype was evaluated as “homozygote genotype” in terms of the risk allele, AT genotype was “heterozygote genotype”, and the TT genotype was “wild type genotype”. As a result of genetic analysis, the frequency of alleles detected in individuals was evaluated according to Hardy-Weinberg equilibrium and genotype frequencies were compared with expected frequencies according to Hardy-Weinberg equilibrium.

### Statistical analysis

The statistical evaluation of the data was performed by using the Windows-based Statistical Package for the Social Sciences (SPSS, version: 22.0) statistical package program. A power calculation conducted a priori using G*Power (version 3.1; Heinrich Heine University Düsseldorf, Germany). The total sample size was calculated as n:124, n: 136, n:156, n:188 for 80, 85 90 and 95% power at the 5% error level, respectively; thus our sample size (n:200) appears to be sufficient for this study.

Count (n), percentage (%), arithmetic mean ± standard deviation (x̅±SD) and minimum(min)-maximum(max) values are given for the measured variables. The χ2 test was used to compare the general characteristics (sociodemographic characteristics, health histories, and physical activity levels, etc.) according to gender and genotypes. The Hardy-Weinberg equilibrium of the gene variants was also analyzed by the χ2 test.

Visual (histogram and probability plots) and analytical methods (Kolmogorov-Smirnov/Shapiro-Wilk tests) were used to evaluate the convenience of data to normal distribution. In the comparison of the anthropometric measurements, adiposity indices and serum insulin, leptin, and ghrelin levels of the participants according to the FTO gene (rs9939609) genotypes, “One Way ANOVA” was performed for the parametric data and “Kruskal-Wallis Variance Analysis” was performed for the non-parametric data. According to the genotypes of individuals, the “Binary Logistic Regression” was performed related to the amount of body fat (%) and it was calculated the odds ratio (OR) and its 95% confidence intervals (CI). Body fat amounts for regression models (%) were classified as “non-high” and “high” by using cut-off values of Lee and Nieman’s gender-based amount of body fat (%) [19]. Further analysis of logistic regression models was performed before and after adjustment for age, gender, and BMI. α:0.05 value was accepted as the level of error in all analyses, and it was evaluated as “the difference between the two groups was statistically significant/important” for *p* values equal to or smaller than this value.

## Results

The study included 100 females and 100 males (39.2 ± 14.01 years) with different BMI values. The sociodemographic characteristics of the individuals who participated in the study were given in Table 1. According to that, 62.0% of the individuals were married while 35.4% of the individuals were single. Most of the individuals were university (45.5%) and high school (26.0%) graduates. 62.5% of individuals had a regular job. The difference between marital status, education level and working status of males and females was statistically significant (*p* < 0.05) (Table 1).

**Table 1:** Evaluation of the sociodemographic characteristics of individuals

	Male (*n*:100)		Female (*n*:100)		Total (*n*:200)			
	n	%	n	%	n	%	χ^2^	*p*
Marital status								
Married	59	59.0	65	65.0	124	62.0	6.995	**0.030**
Single	41	41.0	30	30.0	71	35.5		
Divorced	–	–	5	5.0	5	2.5		
Education Level							13.011	**0.043**
Literate	1	1.0	5	5.0	6	3.0		
Primary school	9	9.0	8	8.0	17	8.5		
Secondary school	13	13.0	8	8.0	21	10.5		
High school	33	33.0	19	19.0	52	26.0		
University	41	41.0	50	50.0	91	45.5		
Postgraduate	3	3.0	10	10.0	13	6.5		
Employment status							6.165	**0.013**
Employed	71	71.0	54	54.0	125	62.5		
Unemployed	29	29.0	42	42.0	75	37.5		

Chi-square test; *p* < 0.05

The FTO gene genotypes and allele frequencies of individuals were shown in Table 2. According to this, when FTO gene allele frequencies of individuals were evaluated, it was determined that 40.2%had A allele and 59.8% had T allele (Table 2). In terms of allele frequency, this situation was found to be 0.4 for A allele, 0.6 for the T allele (This data is not shown in the table). When the frequency of FTO gene alleles is evaluated according to gender, 34.5% of males and 46.0% of females had A allele (Table 2). In terms of minor allele frequency, this was found to be 0.3 for males and 0.4 for females (This data is not shown in the table). 65.5% of males and 54.0% of females have T allele. The difference between FTO gene allele frequencies of male and female subjects was statistically significant (*p* < 0.05) (Table 2).

**Table 2:** Genotype and allele frequencies of the FTO gene according to the gender of individuals

Alleles/Genotypes	Male (n:100)	Female (n:100)	Total (n:200)	χ^2^	*p*
Alleles					
A	69(34.5)	92(46.0)	161(40.2)	5.031	**0.025**
T	131(65.5)	108(54.0)	239(59.8)		
Genotypes				6.504	**0.039**
AA	12(12.0)	26(26.0)	38(19.0)		
AT	45(45.0)	40(40.0)	85(42.5)		
TT	43(43.0)	34(34.0)	77(38.5)		

Genotypes depicted as count (%)

Chi-square test; *p* < 0.05

When the frequency of FTO gene genotypes of individuals is evaluated, 19.0% of individuals were found to have AA genotype, 42.5% have AT and 38.5% have TT (Table 2). The genotype frequency distribution among the total studied population was in Hardy-Weinberg equilibrium (AA: 16.0%; AT: 48.0%; TT: 36.0%) (*p* > 0.05). FTO gene AA genotype frequencies in male and female subjects were 12.0 and 26.0%, respectively. When evaluating the frequency of FTO gene genotypes according to the gender of individuals, it was determined that 45.0% of males have AT and 43.0% have TT genotype. 40.0% of females were determined to have AT and 34.0% have TT genotype (Table 2). When evaluated according to gender, the difference between FTO gene genotype frequencies was statistically significant (*p* < 0.05) (Table 2).

In Table 3, the general characteristics of individuals according to FTO gene genotypes were evaluated. 28.0% of individuals had a chronic disease diagnosed by a physician. Menopause diagnosed in 23.0% of females. 25.5% of them were regularly using medicines and 16.0% are regularly using nutritional supplements. While 46.0% of them were not physically active, 38.0% of them were determined to have low and 16.0% have sufficient activity. According to FTO gene genotypes, the difference between chronic disease, menopause, medication, and nutritional supplements usage and physical activity levels was not statistically significant (*p* > 0.05) (Table 3).

**Table 3:** Some characteristics of individuals according to FTO gene genotypes

	AA Genotype (n:38)		AT Genotype (n:85)		TT Genotype (n:77)		Total (n:200)		χ^2^	*p*
	n	%	n	%	n	%	n	%		
Those with chronic disease	12	31.6	23	27.1	21	27.3	56	28.0	0.294	0.863
Those with a family history of obesity	25	65.8	51	60.0	27	35.1	71	35.5	13.892	**0.001**
Those with menopause ^a^	6	23.1	10	25.0	7	20.6	23	23.0	0.202	0.904
Those who are taking medication	11	28.9	22	25.9	18	23.4	51	25.5	0.427	0.808
Those who are using nutritional supplements	8	21.1	12	14.1	12	15.6	32	16.0	0.956	0.620
Smoker	13	34.2	33	38.8	27	35.1	73	36.5	0.464	0.977
Alcohol consumer	5	13.2	12	14.1	13	16.9	30	15.0	0367	0.832
Physically inactive	15	39.5	38	44.7	39	50.6	92	46.0	3.382	0.496
Physical activity level low	15	39.5	31	36.5	30	39.0	76	38.0		
Physical activity level sufficient	8	21.1	16	15.8	8	10.4	32	16.0		

Chi-square test; *p* < 0.05

^a^n:100

35.5% of individuals had a family history of obesity. When the presence of the family history of obesity according to FTO gene genotypes was evaluated, of the individuals that had AA, AT, and TT genotype, 65.8, 60.0 and 35.1% of their families had a history of obesity, respectively (*p* < 0.05) (Table 3)

The frequency of smoking was 34.2, 38.8 and 35.1% in individuals with AA, AT and TT genotypes, respectively. The frequency of individuals who stated to regularly consume alcohol was 13.2% in individuals with AA genotype, 14.1% in individuals with AT genotype and 16.9% in individuals with TT genotype. The difference between smoking and alcohol use status of individuals according to FTO gene genotypes was not found to be statistically significant (*p* > 0.05) (Table 3).

Anthropometric measurements and adiposity index values of individuals according to FTO gene genotypes were shown in Table 4. When the mean BMI (kg/m^2^) values were compared according to the genotypes of the individuals, the BMI values of those with AT genotypes were 26.3 ± 6.05 kg/m^2^ while those with AA genotype were 24.9 ± 6.00 kg/m^2^ and those with TT genotype were 24.5 ± 6.43 kg/m2(*p* > 0.05) (Table 4).

**Table 4:** Anthropometric measurements, adiposity index values and some hormone levels of individuals according to FTO gene genotypes

Anthropometrics and Adiposity Markers	AA Genotype (n:38)		AT Genotype (n: 85)		TT Genotype (n: 77)		Total (*n*: 200)			
	x̅± SD	Min-Max	x̅±SD	Min-Max	x̅± SD	Min-Max	x̅± SD	Min-Max	Z/t	*p*
Body Weight (kg)	68.1 ± 16.90	37.7–105.6	74.5 ± 17.54	44.6–139.2	70.2 ± 16.15	44.3–113.4	71.6 ± 17.01	37.7–139.2	4.640	0.098 ^b^
BMI (kg/m^2^)	24.9 ± 6.00	16.0–40.7	26.3 ± 6.05	16.3–48.1	24.5 ± 6.43	16.4–44.3	25.4 ± 6.21	16.0–48.1	4.266	0.118 ^b^
Total body fat (%)	28.5 ± 9.25	5.0–44.9	27.0 ± 10.31	6.4–48.7	23.7 ± 10.62	5.0–51.9	26.1 ± 10.61	5.0–58.1	3.253	**0.041**^**a**^
Central fat (%)	38.6 ± 11:03	13.0–54.0	36.2 ± 10.64	17.7–59.0	33.7 ± 10.42	12.3–58.5	35.6 ± 10.68	12.3–59.0	1.747	0.179 ^a^
Internal fat level	11.2 ± 5.83	2.3–24.2	12.5 ± 6.75	2.5–40.5	12.7 ± 7.92	3.0–31.0	12.4 ± 7.10	2.3–40.5	0.335	0.846 ^a^
Waist circumference (cm)	85.9 ± 14.91	59.0–112.09	91.4 ± 14.82	61.0–142.0	87.1 ± 16.94	59.0–132.0	88.7 ± 15.79	59.0–142.0	2.282	0.105 ^a^
Hip circumference (cm)	100.3 ± 11.1	77.5–137.0	102.8 ± 11.63	85.0–136.0	99.2 ± 11.10	75.0–128.0	101.0 ± 11.40	75.0–137.0	3.190	0.203 ^b^
Waist/hip ratio	0.85 ± 0.09	0.69–1.08	0.88 ± 0.07	0.69–1.09	0.87 ± 0.10	0.69–1.29	0.87 ± 0.09	0.69–1.29	1.689	0.187 ^a^
BAI index	29.4 ± 6.82	18.2–49.1	29.4 ± 7.17	16.3–49.3	27.2 ± 7.19	12.2–50.4	28.6 ± 7.15	12.2–50.4	3.865	0.145 ^b^
LAP index	62.0 ± 41.35	12.4–212.4	67.2 ± 41.99	16.5–202.7	61.7 ± 49.2	5.5–226.2	64.1 ± 44.68	5.5–226.2	2.817	0.245 ^b^
Hormones										
Fasting insulin (uIU/mL)	7.0 ± 4.93	1.4–23.7	7.6 ± 5.03	1.3–27.1	6.9 ± 3.21	2.1–19.8	7.2 ± 4.38	1.3–27.1	0.849	0.654 ^b^
Serum leptin (ng/mL)	4.4 ± 5.42	0.03–21.6	4.4 ± 6.05	0.06–29.8	3.4 ± 6.0	0.04–29.8	4.0 ± 5.92	0.03–29.86	5.050	0.080 ^b^
Serum ghrelin (pg/mL)	146.7 ± 124.4	13.6–560.9	114.9 ± 107.58	1.65–474.7	104.5 ± 97.58	6.0–486.7	116.4 ± 107.31	1.65–560.6	3.709	0.157 ^b^

*BMI* Body mass index, *BAI*, Body adiposity index, *LAP*, Lipid accumulation products

^a^ANOVA test, b Kruskal Wallis Test

When the total body fat amount (%) was evaluated according to FTO gene genotypes, total body fat amount of the individuals with AA genotype was high (28.5 ± 9.25%) compared to AT (27.0 ± 10.31%) and TT (23.7 ± 10.62%) genotype (*p* < 0.05). However, there was no difference in central fat amounts (%) (AA: 38.6%, AT: 36.2%, TT: 33.7%) and internal fat levels (*p* > 0.05) (Table 4). Average waist circumference values according to AA, AT and TT genotypes were 85.9 ± 14.91 cm, 91.4 ± 14.82 cm and 87.1 ± 16.94 cm, respectively (*p* > 0.05). Hip circumferences for AA, AT and TT genotypes were 100.3 ± 11.1 cm, 102.8 ± 11.63 cm and 99.2 ± 11.10 cm, respectively (*p* > 0.05). There was no difference in the waist to hip ratios regarding FTO gene genotypes (*p* > 0.05) (Table 4).

When the adiposity indices were evaluated according to genotypes, although BAI and LAP indices were found to be the highest among the individuals with AT and AA genotype, and the lowest among individuals with TT genotype, these differences were not statistically significant (*p* > 0.05) (Table 4).

The levels of fasting insulin (uIU/mL), serum leptin (ng/mL) and ghrelin (pg/mL), which were the food intake and appetite-related parameters according to the genotypes of individuals, were evaluated in Table 4. According to this, no statistically significant difference was determined between the fasting insulin level (uIU/mL) of the individuals with AA, AT and TT genotype (7.0 ± 4.93 uIU/mL, 7.6 ± 5.03 uIU/mL and 6.9 ± 3.21 uIU/mL, respectively) (*p* > 0.05) (Table 4). Serum leptin levels of individuals with AA and AT genotypes (4.4 ± 5.42 ng/mL and 4.4 ± 6.05 ng/mL) were found to be higher than those with TT genotype (3.4 ± 6.0 ng/mL) however, not statistically significant (*p* > 0.05)(Table 4). The mean serum ghrelin levels (pg/mL) of individuals with AA, AT and TT genotype were found to be as follows; 146.7 ± 124.4; 114.9 ± 107.58 and 104.5 ± 97.58 pg/mL, respectively (*p* > 0.05) (Table 4).

Table 5 showed the logistic regression model of the total body fat amount (%) according to the genotypes of the individuals. In the overall population, the probability of the carriers with AT and AA genotypes for being in the group with higher body fat (%) were 2.296 (95% CI: 1.086–4.857) and 2.989 (95% CI: 1.039–8.601) times higher in unadjusted model (*p* < 0.05).

**Table 5:** Logistic regression models (95% CI) for body fat amount (%) in FTO genotypes

	Total Body Fat Mass (%)															
Genotypes	Unadjusted Model				Model 1				Model 2				Model 3			
Overall population	Β	S.E.	OR (95% CI)^a^	p	Β	S.E.	OR (95% CI)^a^	p	Β	S.E.	OR (95% CI)^a^	p	Β	S.E.	OR (95% CI)^a^	*p*
TT	–	–	1	–	–	–	1	–	–	–	1	–	–	–	1	–
AT	0.831	0.382	2.296 (1.086–4.857)	**0.030**	1.076	0.546	2.934 (1.007–8.554)	**0.049**	1.760	0.757	5.811 (1.317–0.286)	**0.020**	1.018	0.835	2.768 (0.539–14.219)	0.223
AA	1.095	0.539	2.989 (1.039–8.601)	**0.042**	0.829	0.382	2.292 (1.083–4.849)	**0.030**	0.006	0.636	1.994 (0.286–3.458)	**0.023**	−0.186	0.673	0.830 (0.222–3.101)	0.782

Body fat amount (%) is classified as “non-high = 0” and “high = 1”

The reference category for genotype is = TT genotype

Β:Regression coefficient. SE:Standart error. BMI: Body mass index

OR: Odds ratio

CI: Confidence interval

Model 1: adjusted for gender

Model 2: adjusted for BMI

Model 3: adjusted for age, gender and BMI

AT and AA genotype significantly increased the probability of being in the group with higher body fat (%) more than two folds with respect to those of wild type (TT) after adjustment for gender in Model 1. After adjustment for BMI in Model 2, the probability of those with AT and AA genotype to be in the group with high body fat amount (%) is 5.811 (95% CI: 1.317–0.286) and 1.994 (95% CI: 0.286–3.458) times higher compared to TT genotype (*p* < 0.05). When the model is adjusted for age, gender and BMI, the probability of AT and AA genotypes carriers related to body fat amount (%) was not found to be significantly different from those with TT genotype (*p* > 0.05)(Table 5).

## Discussion

Obesity is one of the most important public health problems of the twenty-first century. In recently conducted many GWAS, one of the major causes of obesity has been reported to be the FTO gene polymorphism (rs9939609) [7, 8, 10]. In this scope, the association between FTO gene polymorphism and adiposity were evaluated in Turkish adult subjects in this study.

The association of the FTO gene with obesity was first identified in the Caucasian population, and the FTO gene’s SNPs have been shown to be associated with obesity-related parameters such as leptin levels, subcutaneous fat, fat mass, and waist and hip circumference [10]. In some populations, especially rs9939609 polymorphism is associated with obesity, and the A allele (minor allele) has been identified as a risk allele for obesity in this gene [23]. In a large-scale GWAS conducted by Scuteri et al. [12], it was determined that there is a strong relationship between BMI and the FTO gene (rs9930506) in more than 4.000 Sardinians, and FTO is significantly replicated in the people with white Hispanics, or whites and Hispanics race in the United States. There are studies reporting a high prevalence of the FTO gene risk allele in the European population [4, 13, 24]. According to these studies, the frequency of having at least one risk allele in the European population was 50.0–63.0%, while the frequency of homozygous genotypes was reported as 16.0%. It has been reported that FTO risk allele contributes 20% to the obesity cases seen in the Caucasian population [4, 13, 24]. In a study on the Turkish population, the relationship between obesity, metabolic syndrome, and insulin-related parameters of the FTO gene’s rs9939609 and rs1421085 SNPs were investigated in 1967 individuals in the Turkish Adult Heart Disease and Risk Factors (TEKHARF) cohort. As a result, when FTO gene rs9939609 polymorphisms were examined, the AA, AT, and TT genotype frequencies were found to represent 15.7, 48.3, and 36.0% of the population, respectively. Minor allele frequencies were detected as 39.8% for rs9939609-A allele and 42.0% for rs1421085-C allele. However, it was determined that rs1421085 SNP showed linkage disequilibrium and FTO gene rs1421085 SNPs contributed to obesity independently in females and BMI-related metabolic syndrome and insulin resistance in males among Turkish adults [25]. In this study, FTO rs9939609 genotyping of 200 Turkish individuals was performed, and it was detected that 38 (19.0%) of them had the homozygous genotype (AA genotype) for the obesity risk allele, 85 (42.5%) of them had the heterozygous genotype (AT genotype), and 77 (38.5%) of them had the wild-type genotype (TT genotype), and these frequencies were found to be close to the frequencies reported previously in European and Turkish population. In addition, the frequency of A allele, which is a minor allele, in all individuals was found to be 0.4 (Table 2). Genotype frequencies did not differ from those expected in the Hardy-Weinberg equilibrium (AA: 16.0%; AT: 48.0%; TT: 36.0%), and as an important result, the AA genotype was found to be more common in females than in males (26.0 and 12.0%, respectively) (Table 2).

In addition, in this study (Table 3), familial obesity history for individuals with the AA and AT genotypes was found to be higher (65.8 and 60.0%) than that for the individuals with the TT genotype (35.1%) (*p* < 0.05). Likewise, in another study, it was reported that among individuals with the AA genotype, the frequency of familial obesity history in school-aged children was higher, and the familial obesity history was reported to be associated with an approximately 1.2–1.3 times increase in overweight and obesity prevalence in individuals with the AA genotype [26].

In this study, it was determined that there was no difference between the average body weight (kg) and BMI (kg/m^2^) values of the subjects with the FTO gene rs9939609 AA genotype and the individuals with the AT and TT genotypes (*p* > 0.05) (Table 4). In fact, Frayling et al., who discovered this gene, reported that FTO gene rs9939609 polymorphism increased the risk of obesity by 31.0% [4]. In addition, compared to individuals who did not have any risk alleles, the body weights of individuals with homozygous risk alleles were found to be 3 kg (1.5 kg per allele) higher on average; however, they did not have a strong effect on BMI [24]. It was determined that the risk of obesity was 1.67 times higher in individuals with homozygous FTO gene risk alleles, and this relationship was caused by an increase of fat mass specifically from the age of 7 years [4]. In another study, it was determined that FTO rs9939609 polymorphism was associated with an increase in the BMI of 0.42 units, in body fat percentage of 1.03%, and in waist circumference of 0.85 cm per allele [27]. Since it was determined in the literature that different SNPs show strong attachment inequalities in each population, it is not yet clarified which variant shows a major effect on BMI and thus obesity [24]. Similar to the extant literature, this study presumes that the reason that FTO gene rs9939609 polymorphism does not have a significant increasing effect on BMI may be due to racial differences. Thus, in the Turkish population, different FTO SNPs may be more effective on BMI than FTO rs9939609 polymorphism. Further studies with large samples will be more enlightening in this regard.

FTO shows a high level of expression in brain, adipose, and muscle tissue development and/or adulthood. Adipose tissue is the region where excess energy is stored, and the differentiation and proliferation of preadipocytes play a key role in obesity. Therefore, the different levels of FTO expression in populations with different BMIs are considered as other obesity-related mechanisms. Studies have shown that there is a positive relationship between FTO mRNA level and BMI in subcutaneous adipose tissue [28, 29], and FTO mRNA levels were higher in adipose tissue in obese subjects [30, 31]. In another study, it was determined that FTO knockdown causes suppression of preadipocyte proliferation, lower mitochondrial membrane efficiency, lower cellular adenosine triphosphate (ATP), and fewer and smaller lipid particles [28].

In this study, it was determined that regardless of BMI, the total body fat amount (%) of the individuals with the A allele, which is a minor allele, was 3–5% greater than in those without an A allele (*p* < 0.05) (Table 4, Table 5). Significance of association between FTO genotypes and total body fat (%) was retained after adjustment for BMI (Table 5). There was no difference in central fat accumulation, internal fat level, or waist to hip ratios (Table 4). This study, as far as is known, is the first study to evaluate the abdominal region fat levels via infrared analysis method in terms of genotypes. In light of these results, it can be said that FTO rs9939609 polymorphism is associated with fat accumulation in the whole body without being associated with abdominal fat accumulation. On the contrary to the present study, some studies showed that FTO variants are significantly associated with distribution of fat in some populations [32, 33]. A study found that FTO genotype (rs9939609) was associated with body weight in general, whereas adrenoceptor beta 2 (ADRB2) genotype was associated with fat distribution in obese women [34]. Another study found that there was a significant effect of variation in the FTO gene (rs8050136) was associated with a higher BMI, body fat, and also subcutaneous fat & visceral fat in 1466 German subjects [35]. Effect of FTO gene polymorphism on body fat and its distribution is not clear due to racial differences.

Another important result of study that FTO genotypes and total body fat (%) was associated independently gender (Table 5). Interestingly, there are some studies that have determined the relationship between FTO rs9939609 polymorphism and obesity based on BMI in females only [34, 36–39]. This relationship was revealed more clearly in females than in males in non-Hispanic Caucasian [36], Portuguese [37], Cebu Filipino [38], Finnish [34], and Australian [39] populations. These results may be due to the fact that it was not shown differences in BMI between genders according to FTO genotypes in the present study.

In this study (Table 4), it was determined that LAP and BAI index values, which are used in body fat estimation, were not different between different genotypes in all individuals and in different genders (*p* > 0.05). Some studies have shown that the FTO gene is often associated with adiposity indices such as LAP and BAI in individuals with chronic disease or in the postmenopausal period [40, 41]. Of the patients with hypertension, those with FTO rs9939906 AA/AT genotypes were associated with higher LAP and BAI index values [40]. In postmenopausal females, the LAP index of those with FTO rs9939609 and rs8050136 AA genotype was found to be higher [41]. The results obtained in this study are thought to be mostly due to the fact that the sample group was generally composed of healthy individuals, and the anthropometric measurement values used in the calculation of these indices do not differ according to genotypes.

In this study, the relationship of the FTO gene with hormones that are involved in food intake was also evaluated (Table 4). Expression of the FTO gene in the hypothalamic nucleus, which plays a key role in energy metabolism, suggests that the FTO gene contributes to energy homeostasis [13]. It has also been proven that the level of mRNA of FTO is regulated according to fullness and fasting [5, 42]. Gerken et al. [5] found that expression in the hypothalamus of mice increased by about 60% in fullness compared to the fasting state. They reported that genetic variations in the loci of the FTO gene (rs9939609, rs17817449, and rs142085) contribute to the aetiology of obesity by affecting the level of plasma leptin and causing insulin resistance [14]. In another study, it was reported that the mRNA level of acyl ghrelin was higher in individuals with the FTO gene AA genotype compared to individuals with the TT genotype [15]. Furthermore, excessive expression of FTO in cell models has been reported to reduce ghrelin mRNA m^6^methylation and simultaneously increase ghrelin mRNA and peptide levels [15]. In another study, the plasma adiponectin and leptin levels of 854 diabetic males and 987 diabetic females were evaluated, and rs9939609 SNP in diabetic females was detected to be associated with lower adiponectin (1.82 ± 0.04, 1.73 ± 0.03, and 1.68 ± 0.05 for TT, AT, and AA genotypes, respectively) and leptin levels (3.56 ± 0.04; 3.63 ± 0.04 and 3.70 ± 0.06 for TT, AT and AA genotypes, respectively) [43]. In individuals with homozygous risk alleles, it has been determined that this decreases the insulin response by affecting cerebrocortical insulin sensitivity [44]. In this study, there was no difference in the fasting insulin (uIU/mL), serum leptin (ng/mL), and ghrelin (pg/mL) levels of individuals with the different FTO rs9939906 genotypes (*p* > 0.05) (Table 4). These results may be due to the fact that this study was performed on non-diabetic individuals. Also, in this study, the expression level of the FTO gene was not evaluated according to fasting-fullness situation. In the evaluations to be performed that are directed at individuals’ appetites, it may be useful to evaluate the expression levels of FTO in future studies.

In many GWASs in the literature, besides the determination of the frequencies of FTO genotypes, the expression levels were also investigated, and an increased expression level was found to increase the risk of obesity by affecting these parameters [45, 46]. In addition, it was reported that the FTO gene realizes its effect on body composition and obesity in interaction with other genes, such as FTM (RPGRIP1L) and Irx3, which are localized near it [46]. Therefore, in future studies to evaluate the effect of the FTO gene on body composition, obesity, and appetite, it is important to evaluate the level of expression and the interaction with other genes.

## Conclusions

In conclusion, variation within the *FTO* locus (rs9939609) was associated with total body fat even after adjustment gender and BMI. However, there was no difference in abdominal fat, adiposity indexes such as LAP and BAI, or some appetite markers, such as insulin, serum leptin, and ghrelin. This study, as far as is known, is the first study to evaluate the abdominal/central fat status through the infrared analysis method according to FTO (rs9939609) genotypes.

However, the present study has a number of limitations. First, a single locus of the FTO gene (rs9939609) was searched in the present study. Other loci of the FTO gene may have different effects on body composition. Second, we didn’t evaluate the mRNA expression levels of FTO (rs9939609) genotypes and appetite/hormonal markers. Alterations of mRNA expression level of the FTO gene and hormonal factors can play important roles in body composition. Third, the number of samples was relatively low compared to some large-population studies because we used an infrared analysis method to determine central fat, and this method required specific measurements and environmental conditions. Moreover, it must be noted that the studied population did not include a significant number of those with morbid obesity, and the majority had a BMI of less than 30 kg/m^2^, which is one of the limitation in this study. It is believed that taking these situations into consideration would be useful in future studies.

## Data Availability

We declare that the data supporting the conclusions of the article are fully described within the article, and the database is available from the first author (duyguturkozu@gazi.edu.tr) upon reasonable request.

## Abbreviations

ADRB2: Adrenoceptor beta 2
ATP: Adenosine triphosphate
BAI: Body adiposity index
BHQ: Black hole quencher
BIA: Bioelectrical impedance analysis
BMI: Body mass index
CI: Confidence interval
DNA: Deoxyribonucleic acid
EDTA: Ethylenediamine tetra acetic acid
ELISA: Enzyme-linked immunosorbent assay
FTO: Fat mass and obesity-associated
GWAS: Genome-Wide association studies
IPAQ-SF: The International Physical Activity Questionnaire - Short Form
LAP: Lipid accumulation products
MET: Metabolic equivalents
mRNA: Messenger RNA
OR: Odds ratio
PCR: Polymerase chain reaction
RNA: Ribonucleic acid
SNP: Single nucleotide polymorphism
SPSS: Statistical package for the social sciences
TEKHARF: Turkish Adult Heart Disease and Risk Factors
TG: Triglyceride
WHO: World Health Organization
